# The Role of Perceived Social Norms in Rural Sanitation: An Explorative Study from Infrastructure-Restricted Settings of South Ethiopia

**DOI:** 10.3390/ijerph14070794

**Published:** 2017-07-17

**Authors:** Josef Novotný, Jana Kolomazníková, Helena Humňalová

**Affiliations:** Department of Social Geography and Regional Development, Faculty of Science, Charles University, Prague 12843, Czech Republic; kubelkova.jjana@seznam.cz (J.K.); helena.humnalova@natur.cuni.cz (H.H.)

**Keywords:** Ethiopia, CLTS, open defecation, sanitation, social norms, toilets

## Abstract

The perception of social sanitation norms (PSSNs) around unacceptability of open defecation has been a key aspect of recent sanitation interventions. However, underlying mechanisms through which “reconstructed” PSSNs affect sanitation outcomes have been a black box. This explorative cross-sectional study examines direct and indirect links between PSSNs and sanitation safety using data from structured interviews and observations in 368 households in rural South Ethiopia. In addition to a positive association between PSSNs and sanitation safety, we propose and examine the following two mechanisms: First, we confirm a potentially adverse feedback of PSSNs on future sanitation safety by enhancing the emotional satisfaction with current sanitation practice (satisfaction independent of the functionality of sanitation facilities). Second, inspired by the social amplification/attenuation of risk framework, we demonstrate that PSSNs work as a “social filter” that can amplify or attenuate the effects of other variables targeted in sanitation interventions such as perceived health-related and non-health risks and benefits associated with open defecation and private latrine ownership, respectively, and factual hygiene and sanitation knowledge. These findings imply that PSSNs are not only important per se, but they are also important instrumentally because sanitation outcomes depend upon the capacity of social influences to shape the perception of sanitation risks and benefits and sanitation-related awareness in desirable ways. The mechanisms outlined in this paper as well as the sustainability of sanitation outcomes depend on whether and how social sanitation norms are internalized.

## 1. Introduction

**Figure 1:**
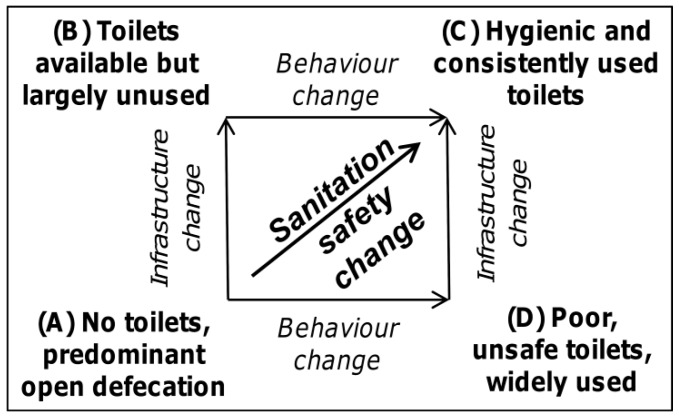
Different pathways to sanitation safety. Source: Produced by the authors.

Improving sanitation safety by eliminating open defecation (OD) and increasing the access and utilization of toilets that hygienically separate human excreta from human contact can bring important health benefits [1,2,3,4] and is also known to generate notable socio-environmental transformation [5,6,7,8,9]. Yet, it is estimated that in 2015 2.4 billion people lacked access to a safe sanitation facility and that nearly one billion people still defecate in the open with the majority living in the countries of Sub-Saharan Africa and South Asia, often in environments characterized as infrastructure-restricted [10]. Efforts to improve latrine coverage and/or use consist of interrelated hardware (sanitation technology) and software (sanitation behavior) dimensions. Accordingly, different paths from predominant OD to a sanitation-safe environment exist (Figure 1). Interventions focused predominantly on providing or subsidizing latrine construction, but neglecting issues related to various social, cultural and environmental constraints of their consistent usage are known to pose a risk of the “A”→“B” path in Figure 1, signified by the disuse of new sanitation facilities or recurrence of OD practice [11,12,13]. On the other hand, interventions aimed predominantly at the shift of sanitation behavior from OD to fixed-point defecation but disregarding the questions of functionality and durability of sanitation facilities may result in the “A”→”D” path in Figure 1.

Traditional campaigns to change sanitation behavior focused primarily on creating awareness and communicating health risks through education and information promotion often delivered at the household level [14]. Factors other than awareness of health risks and benefits have nevertheless been increasingly reported as more important drivers of people’s demand for improving their sanitation safety. These include prestige and the will to adopt a modern lifestyle [5], wealth, privacy, comfort, security [15], social networks, social expectations and power relations [9,16,17,18], locally specific taboos or cultural factors [19,20,21,22], among other factors. It has also been acknowledged that sanitation safety represents a specific type of private–public goods in the sense that benefits occur only when an entire community changes its sanitation practice. Consequently, approaches focused on social and emotional factors to change perceived social sanitation norms (PSSNs) at a community level have gained in popularity.

The Community-Led Total Sanitation (CLTS) has become a particularly popular sanitation strategy used in more than 60 countries across the world (see www.communityledtotalsanitation.org). It uses participatory techniques to facilitate communities’ self-appraisal of their local environment, heighten the perceived benefits of latrine use, provoke emotions such as disgust, shame and fear of illness to create a sense of peer pressure, and change PSSNs to establish OD as being socially unacceptable [13,14,23,24]. CLTS is reportedly effective in its main goal of eliminating OD [25,26,27,28,29,30]. Nevertheless, it poses a risk of the A”→”D” path in Figure 1 as CLTS campaigns often result in nondurable and unsafe latrines [27,28,29,31,32] so their longer term impacts are still open to debate. Moreover, some CLTS critiques have raised concerns regarding the use of unethical practices such as shaming, stigmatizing, and punishing community members [33,34], and the risk of mechanistic application [35]. Despite the controversy, CLTS provided the ground-breaking revelation that newly reconstructed PSSNs drive sanitation behavior [36]. Correspondingly, the importance of PSSNs has been acknowledged in theoretical accounts of sanitation safety change [37,38] and formative research into PSSNs has been recommended to practitioners [37,38,39]. However, no previous research has empirically analyzed how PSSNs instilled through CLTS or other persuasive/normative interventions affect sanitation outcomes.

This paper provides an explorative study that examines this question using cross-sectional data collected in 2015 in rural South Ethiopia using structured interviews and direct observations in 368 households. Ethiopia represents a particularly interesting context for this exercise because an Ethiopian adaptation of CLTS (referred to as the Community-Led Total Sanitation and Hygiene—CLTSH) has been integrated into the country’s national sanitation strategy [40] and facilitated through a dense network of health extension workers (HEWs). This can arguably be considered a major factor behind Ethiopia’s fast reduction of OD rate from 92% in 1990 to 29% in 2015 [10,41]. However, poor quality toilets and recorded returns to OD [42] interrelated with inadequate attention to sanitation infrastructure indicate that the change might be unsustainable. More nuanced understanding of the role of socially constructed PSSNs is thus warranted.

**Figure 2:**
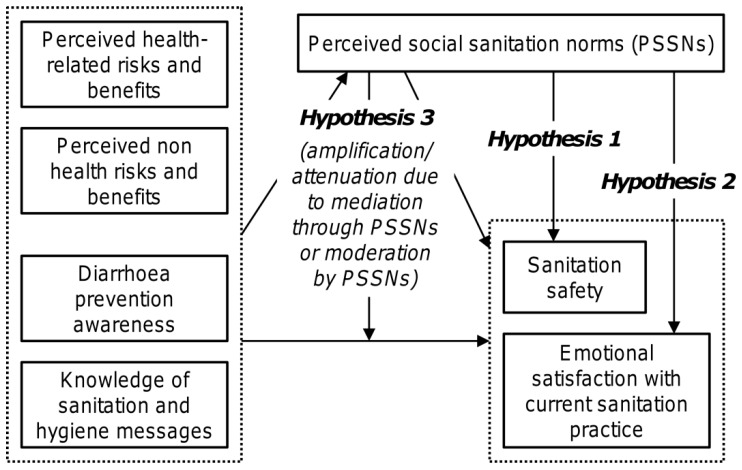
Hypotheses examined in this study (H1—direct effect of PSSNs on sanitation safety; H2—effect of perceived social sanitation norms (PSSNs) on emotional satisfaction; H3—PSSNs moderate or mediate effects of perceived risks and benefits and hygiene and sanitation awareness).

Figure 2 schematically outlines three hypotheses described below and examined in this study. The first one represents a conventional view that considers PSSNs to be a strong direct determinant of sanitation safety (Hypothesis 1). However, we additionally assume that PSSNs may also influence sanitation safety through other routes. As the second hypothesis, we propose that an indirect effect may lead through the effect of PSSNs on one’s emotional satisfaction within the current sanitation situation. Unlike material satisfaction driven by latrine performance and attained sanitation safety, emotional satisfaction is independent of the attained level of sanitation safety and can emerge because of social conformity itself. Emotional satisfaction is known to be associated with false positives [43]. Therefore, in this context, it would mean a potentially adverse feedback effect undermining further upward shifts in the sanitation ladder. This may be an issue especially when a new fixed-point sanitation practice is introduced through persuasive/normative interventions which place the effect of social conformity at the centre in infrastructure-restricted settings characterized by the generally low quality and non-durability of sanitation facilities. Accordingly, the second hypothesis examined in this paper thus analyzes the relationship between PSSNs and the degree of satisfaction with one’s current sanitation practice when the level of current sanitation safety is controlled (Hypothesis 2).

Another important question is how PSSNs affect (interplay with) other determinants of sanitation safety. In this respect, perceived risks and benefits associated with OD and private latrine ownership, respectively, and factual knowledge related to hygiene and sanitation represent particularly interesting types of factors that are also typically targeted by various sanitation campaigns. We argue that the social amplification/attenuation of risk framework [44,45] can be a useful interpretative framework for our third hypothesis which proposes non-trivial interactions between PSSNs and the perceived risks and factual knowledge in their joint effects on both sanitation safety and emotional satisfaction (Hypothesis 3). Although it has not been used in the area of sanitation so far, the Social amplification/attenuation of risk framework has already been applied in various areas dealing with interplays between the technical aspects of risks and individual, social, cultural, and institutional structures that influence the public response to these risks. It portrays risk information as signals that can be amplified during their transmission if they appear frightening enough, or attenuated if they are less threatening [45,46]. It emphasizes the reflexive character of social processing of risk information in which social parameters interact with cognitive-educational variables and affects behavior accordingly. Importantly, social amplification/attenuation of risk is most likely to apply for risks and events where cause and effect relationships are not straightforward, for example, because the effects are delayed in time or due to complex underlying mechanisms that make the isolation of cause and effect links difficult [45]. This holds well for sanitation interventions where their possible positive health effects are known to be delayed [47], conditional to whether behavior change involves an entire community, confounded, and generally difficult to measure [48,49] and thus also uneasy to trace by laypeople. The metaphor of social amplification/attenuation can also be especially relevant when sanitation-related threats are perceived as comparatively less urgent than other more obvious problems affecting local people. In such contexts, the processes of social amplification become an important aspect of sanitation campaigns with the results depending on whether and how effectively the right aspects of sanitation-related risk and awareness information is transmitted through the targeted community. As such, PSSNs can then play an important moderating or mediating role in transmission processes.

## 2. Materials and Methods

### 2.1. Data

This study draws on data collected in September and October of 2015 in 11 kebeles (smallest administrative units in Ethiopia) of two woredas (districts), namely Kindo-Koysha and Diguna Fango woredas, in the Southern Nations, Nationalities, and Peoples’ Region of Ethiopia. These districts represent exemplar cases of sanitation infrastructure-restricted settings and met our aim to survey communities in diverse environmental conditions (high elevational heterogeneity associated with substantial differences in agro-climatic conditions and accessibility) but similar institutional environments. To cover diverse communities, the surveyed kebeles were selected randomly from three sub-groups of kebeles determined based on their travel accessibility, geography (low-, mid-, high-land), and protected drinking water availability. The survey consisted of structured interviews and direct observations of latrines and their surroundings in households (*N* = 368). The data collection was administered by five experienced data collectors in the local language (Wolaytta). A random walk technique was used to sample households within the kebeles with an effort to reflect their spatial structure. Heads of households were preferentially interviewed; if the household head was not present another adult member (preferably spouse) was interviewed. Interviews lasted for an average of 40 min and consisted of 86 questions about demographics, socioeconomic information, subjective health and health risks, sanitation and hygiene awareness, self-reported sanitation behavior, sanitation infrastructure, PSSNs, social interactions, social capital, and water availability. An additional 14 parameters related to latrine and surroundings were assessed by direct observations of latrines and other sanitation infrastructure. Semi-structured interviews with HEWs and community leaders (*N* = 20) in the surveyed kebeles were also conducted but this material has only a supplementary role in this paper.

Our research received approval from the Ethiopian authorities and was approved by the institutional ethical committee of Charles University (approval number 2015/32). All participants and informants participated in the study voluntarily, providing free and informed consent while being assured of anonymity and confidentiality. A collaborating non-governmental organization (NGO), People in Need (PiN) played a consulting role in respect to the design of our survey with no involvement in data analysis and interpretation.

### 2.2. Measures

The main dependent variable of sanitation safety is a composite index determined using the categorical principal component analysis based on 11 characteristics of availability, functional quality, and the utilization of sanitation facilities identified using a reliability analysis from more potentially relevant parameters. The Cronbach’s Alpha of 0.749 suggested an acceptable level of internal consistency. The 11 characteristics captured (1) the availability of a private latrine, (2) whether the latrine is not apparently unused, (3) whether it has a solid slab platform, (4) whether it has a solid superstructure, (5) whether it ensures a basic privacy, (6) whether it does not smell aggressively, (7) whether it has a hole cover, (8) whether the pit is covered, (9) whether water, ash, or soap is available for handwashing, (10) whether the latrine and house surroundings is clean of human feces, (11) whether there are no animal feces in front of the house, and (12) whether OD is reported as prevalent practice in at least one time or season (i.e., day-time, night-time, rainy-season, dry-season).

The second dependent variable was a dichotomous measure of respondents’ satisfaction with their current sanitation practices. Here, respondents were asked to consider their overall satisfaction with both hardware and behavioral aspects of the current sanitation situation in their households.

To measure PSSNs, eight questions or statements focused on the perception of descriptive and injunctive social norms related to sanitation were elicited. We gauged respondents’ perceptions about whether other members of their village defecate in a latrine, about the sanitation behavior of people important to them, their normative views on the behavior (what they believe others ought to do), how they believe their community perceives their sanitation practices, and their beliefs about their community’s normative views of others regarding sanitation practice (what others think one should do). After examining the consistency of particular items, six of them that are shown in Table 1 were selected with their Cronbach’s Alpha of 0.780 and used to determine a composite score of PSSNs. No significant difference was found regarding the consistency of items on descriptive and injunctive norms so items about both of these aspects were used for the construction of the single composite measure of PSSNs. All questions and statements were originally measured using the five-point Likert scales. The reported unacceptability of OD was generally high, though by far not ubiquitous (the second column of Table 1). Given the skewed distributions of responses, particular items were binarized before running the categorical principal component analysis to obtain the aggregate measure of PSSNs.

The perceived risks and benefits related to sanitation were measured using two open questions concerning the risks or disadvantages associated with OD and the advantages or benefits of having a private latrine. Responses were classified into a few predefined categories of risks and benefits. These types of risks and benefits were largely symmetric in the sense that analogous types of advantages of private latrine and risks or disadvantages of OD were reported. Two variables of perceived risks and benefits were then constructed by distinguishing health-related risks and benefits (up to six categories related to human and environmental health) and the perceived non-health risks and benefits (up to nine categories including privacy, security, cleanliness, convenience of use, prestige and social respect, etc.).

Factual sanitation-related knowledge was measured by two variables. The first was a binary variable constructed through an open question about the most effective ways of preventing diarrhea in order to distinguish between those with no diarrhea prevention awareness, and those with some or good awareness. The second measure was constructed through an open question about the awareness of hygiene and sanitation messages, quantifying the sum of relevant messages reported by respondents (up to eight messages). Table 2 contains basic descriptive statistics of the focal measures described above.

A number of other variables capturing household- and individual-level diversity in our sample were also assessed. Table A1 in Appendix A shows a selection of these descriptive characteristics of the sample that were scrutinized as potential control variables in further analyses.

### 2.3. Data Analysis

**Figure A1:**
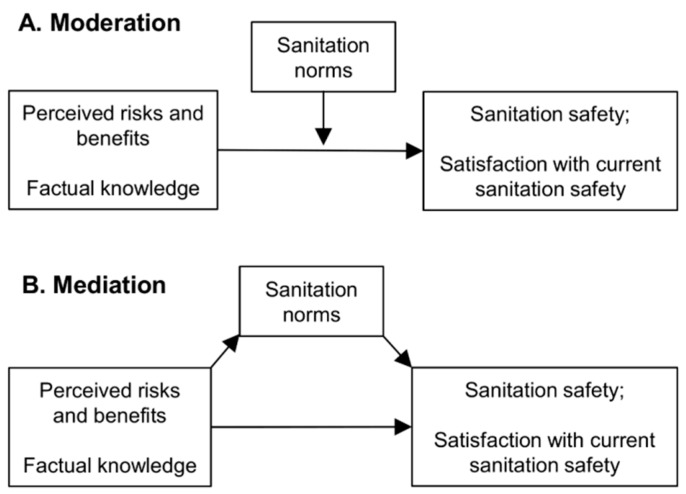
Conceptual diagrams of moderating and mediating role of social sanitation norms (Hypothesis 3). While moderation (**A**) focuses on how the effect of perceived risks and benefits or factual knowledge changes for different levels of sanitation norms, mediation analysis (**B**) examines an indirect effect of perceived risks and benefits or factual knowledge through sanitation norms.

The sanitation safety index, which is considered to be the first dependent variable, revealed a distribution that was sufficiently close to normal distribution, so linear regression models were used to analyze its predictors. Binary logistic regressions were applied to model the second dependent variable of satisfaction with current sanitation practices. Due to concerns about data clustering at the kebele level, mixed regression models were firstly examined but the kebele-level random effects were not significant. Fixed effects models with kebele-level controls were thus used. Continuous variables were standardized by z-scores and appropriate checks and transformations were undertaken to correct for outliers. Despite some correlations between our focal variables, collinearity statistics were acceptable for all of the examined models. Models examining only main effects of independent variables (i.e., with no interaction terms) were firstly run with the focal variables of interest and relevant control variables (various sets of potentially relevant control variables from Table A1 in Appendix A were examined). Comparisons of models with and without PSSNs indicated the influence that PSSNs yield on the regression coefficients of the measures of perceived sanitation-related risks and benefits, and measures of factual hygiene and sanitation knowledge. This was also instrumental for examining our third hypothesis about the possible moderating and mediating role of PSSNs invoked on the basis of the social amplification/attenuation of risk framework. While moderation focuses on how the effect of an independent variable changes for different levels of a moderator variable, mediation analysis can help to uncover an indirect effect of an independent variable through a mediator variable (conceptual diagrams appear in Figure A1 in Appendix A). The potentially moderating role of PSSNs was firstly explored through a consecutive examination of particular two-way interaction terms between PSSNs and other focal independent variables. In the next step, preconditions of possible mediation through PSSNs were explored and when in place, the presence of an indirect effect was tested in the way described in [50].

## 3. Results

### 3.1. Contextualization

Already high socioeconomic and environmental vulnerability of the surveyed rural communities were further exacerbated by the exceptional 2015 El Niño drought at the time of the survey. Food shortages were clearly perceived as the most serious threat and health risk, while threats and health risks related to hygiene and sanitation were perceived as comparatively minor. This indicates the applicability of the social amplification/attenuation of risk framework in this context as well as the fact that mechanisms other than direct motivation to improve health would play a key role in attempts to improve household sanitation safety.

**Figure 3:**
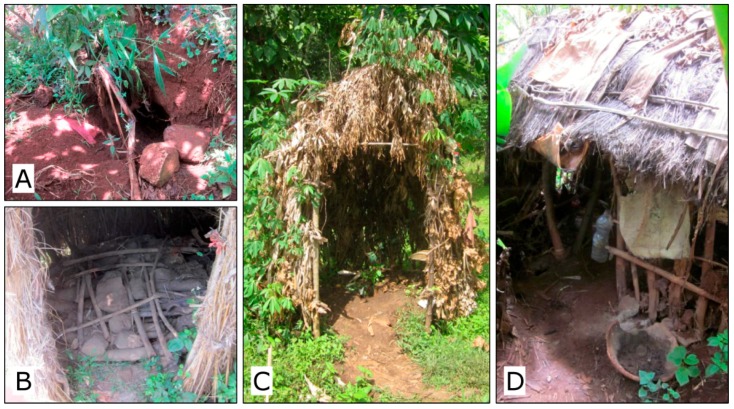
Examples of surveyed sanitation facilities: (**A**)—Unimproved pit, no other facilities; (**B**)—Simple slab platform, squatting hole covered; (**C**)—Pit latrine with solid slab, basic superstructure, apparent footpath; (**D**)—Pit latrine with solid slab, superstructure ensuring privacy, water and ash available for handwashing.

The majority (89%) of households in our sample had a private pit latrine, while most of those without a latrine reported that they had one in the past. All of the toilets were simple pit latrines (Figure 3), however, only 47% of them met the World Health Organization/United Nations Children's Fund (WHO/UNICEF) definition of an improved sanitation facility. Despite a generally low quality of sanitation facilities, the majority of respondents (72%) reported that they are satisfied with their current sanitation practices, and the satisfaction was still high (68%) among those with unimproved latrines. The latrines were almost solely made of local materials. Neither commercial vendors of sanitation infrastructure nor external supply-side sanitation interventions were present in the surveyed villages that would provide more durable sanitation products and services. Correspondingly, only very few respondents indicated financial costs among the disadvantages of latrine or among the reasons for not improving the latrine. Also, for those without any latrine, financial constraints were considerably less of an issue than a lack of materials or a shortage of manpower.

According to information we received in the districts’ health centers, an identical sanitation approach has been used in the surveyed villages that generally corresponded to the official guidelines for rural sanitation. The CLTSH represented a major component together with other awareness activities mainly delivered by HEWs who have served as the local agents under the Health Extension Program (introduced in 2004). Importantly, the Southern Nations, Nationalities, and Peoples’ Region, where our survey was conducted, was the first Ethiopian region where a sanitation program has been implemented already since 2003. Accordingly, 72% of respondents reported that their first latrine was constructed five or more years ago. Although an identical sanitation approach has officially been used, notable differences are likely to exist between particular kebeles with respect to its implementation due to differences in institutional support and the capacity and workload of HEWs in particular villages (as indicated in the interviews with HEWs and village leaders). Although all of the surveyed villages were officially recorded as open-defecation-free villages by district health centers, in at least three cases this was not true at the time of our survey according to local HEWs. Importantly, in most of the communities, formal and semi-formal sanctions for the absence of a latrine (introduced following the CLTSH campaigns) were reported to still exist at the time of our survey. Such sanctions reportedly included public shame and ridicule at community meetings, and the threat of fines or short-term jail sentences.

There were statistically significant differences in the village-level averages for some of the focal variables analyzed in this paper. Although the village-level fixed effects were included into the regression models presented below, it is notable that the variation between villages was most pronounced for the factual hygiene and sanitation knowledge, perceived non-health risks and benefits, and sanitation safety score. The abovementioned differences in the implementation of sanitation campaigns and related activities of HEWs may provide an explanation. Interestingly, however, the measures of PSSNs, satisfaction with current sanitation practice, and perceived health risks and benefits were not significantly different across the surveyed villages. The observation that these variables are independent of the village-level specifics is important because these three variables (and their interplay) have a central role with regards to the second and third hypothesis examined in this paper.

### 3.2. Regression Analyses

**Figure 4:**
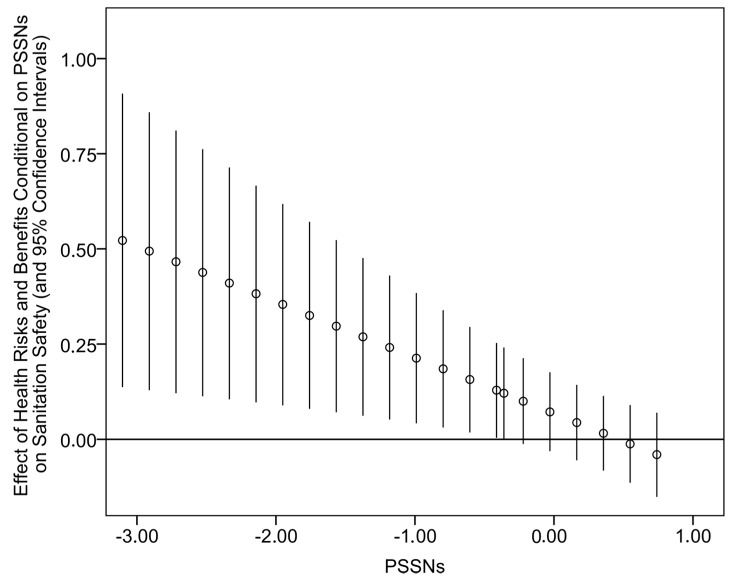
Effects of perceived health-related risks and benefits on sanitation safety conditional on different values of PSSNs (small circles) and their 95% confidence intervals.

The regression results for the predictors of sanitation safety appear in Table 3. For the sake of readability, Table 3 only contains regression estimates for our focal independent variables. The full specification of the main effects model with estimates for control variables can be found in Appendix A, Table A2. The results presented in Table 3 show that the considered predictors of sanitation safety explain around 40% of variation, which is a good explanatory power given the assumed complexity of sanitation safety determinants. Importantly, a great deal of this variation can be attributed to our focal independent variables (we noted that the main effect model without the focal independent variables explained only around 15% of variation). Congruently with Hypothesis 1, PSSNs were identified (*p* < 0.01) as the comparatively strongest predictor of sanitation safety. The main effects of other focal independent variables were also positive and statistically significant with the exception of perceived health-related risks and benefits. The examination of separate regression models that included particular two-way interactions between PSSNs and other independent variables revealed that PSSNs moderate the effect of perceived health-related risks. The interaction term was negative (*p* < 0.01), and a more nuanced look at this conditional relationship showed that it is statistically significant for 27% of all observations with the level of PSSNs below 0.407. A graphical visualization of the conditional relationship appears in Figure 4. Despite the restricted region of significance, these results suggest that PSSNs tend to buffer the (already low) influence of the perception of health-related risks and benefits on the level of sanitation safety of households.

**Figure 5:**
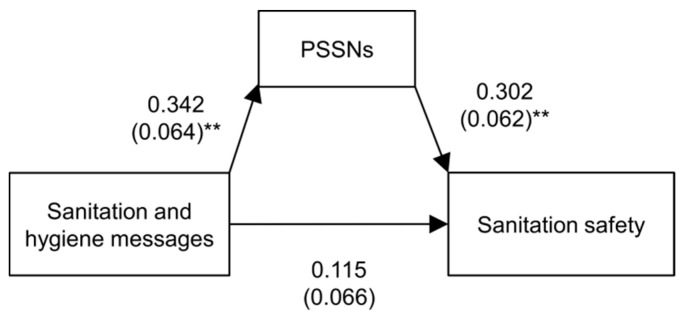
Mediation of PSSNs between the knowledge of sanitation and hygiene messages and sanitation safety. Beta coefficients and standard errors (in parentheses). ** Significant at the 0.01 level.

The results compared for main effect models with and without PSSNs (second and third column of Table 3) show that the effect of the factual knowledge of sanitation and hygiene messages was statistically significant (*p* < 0.01) only when we excluded PSSNs. This is consistent with the mediation model. Other tests also suggested that PSSNs mediate between the factual knowledge of sanitation and hygiene messages and sanitation safety. The estimated indirect effect corresponds to 0.103 (*p* < 0.01). A schematic graphical representation of the mediation appears in Figure 5. Importantly, these results indicate that the link between the knowledge of sanitation and hygiene messages and sanitation safety becomes identifiable only when persuasive power of social norms is taken into account.

**Figure 6:**
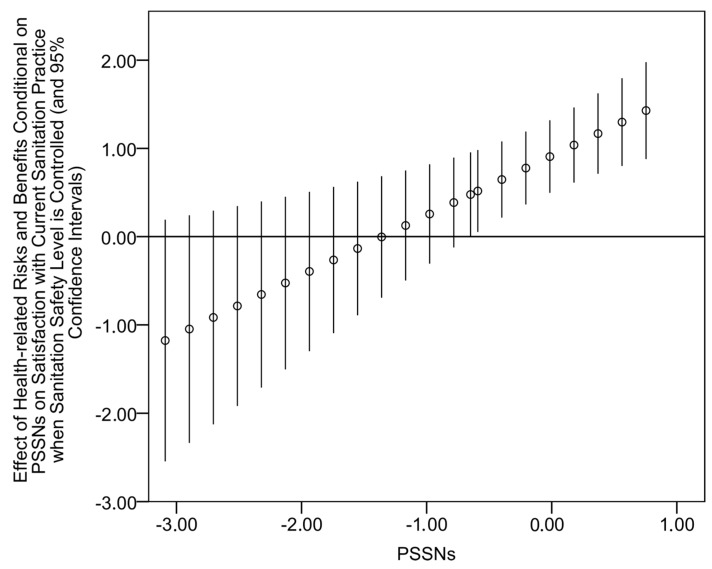
Effects of perceived health-related risks and benefits on satisfaction with current sanitation practice when sanitation safety is controlled conditional on different values of PSSNs (small circles) and their 95% confidence intervals.

**Figure 7:**
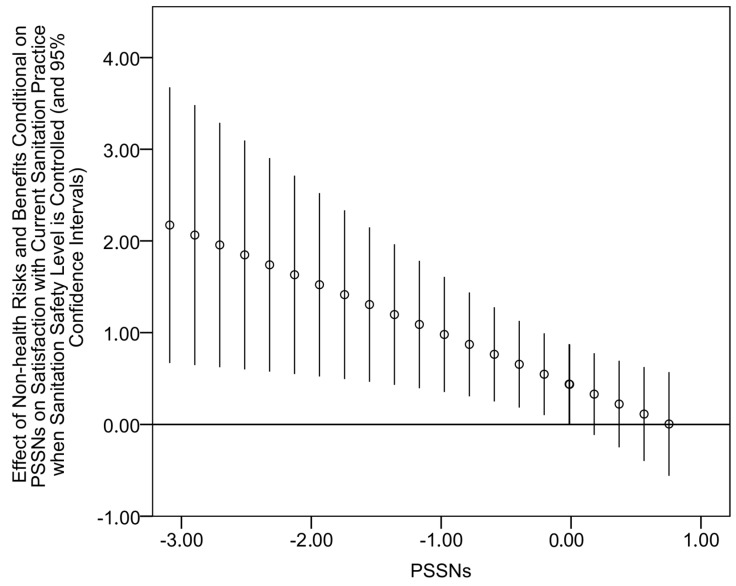
Effects of perceived non-health risks and benefits on satisfaction with current sanitation practice when sanitation safety is controlled conditional on different values of PSSNs (small circles) and their 95% confidence intervals.

Table 4 presents results for the predictors of satisfaction with current sanitation practice (the full specification of the main effects model with estimates for control variables can be found in Appendix A, Table A3). It also reports regression estimates for the measure of sanitation safety. Although here it was primarily considered as a control variable, its respective beta coefficient is high and positive (*p* < 0.01) which confirms the importance of attained sanitation safety for satisfaction with current sanitation practice. Unlike other independent variables of interest, PSSNs and perceived health-related risks also revealed significant positive effects on satisfaction with current sanitation practice (*p* < 0.01) when the level of sanitation safety was held constant. It suggests that these two variables enhance the emotional aspect of satisfaction. Moreover, their mutual interaction term was also statistically significant (*p* < 0.01) and positive. More specifically, the conditional effect was statistically significant for 79% of observations with PSSNs above −0.641 (Figure 6). These results mean that PSSNs tend to amplify the effect of perceived health-related risks and benefits on emotional satisfaction with current sanitation situation. By contrast, the interaction between PSSNs and perceived non-health risks and benefits was negative suggesting that PSSNs weakens the effect of the latter. This conditional effect was significant at the 95% level for observations with PSSNs below −0.020 (Figure 7). Finally, two variables of factual sanitation-related knowledge were found unrelated to satisfaction with current sanitation practices.

## 4. Discussion

The results obtained in this study are consistent with all three hypotheses outlined in the introduction (Figure 2) and they generally show that the relationship between PSSNs and sanitation safety of households is more ambiguous than commonly thought. Congruently with Hypothesis 1, a significant association between PSSNs and sanitation safety was confirmed. However, other indirect routes through which PSSNs can affect sanitation safety have been outlined too. As per Hypothesis 2, PSSNs were found to be positively related to satisfaction with current sanitation practices when the level of sanitation safety was controlled. We interpret this finding as implying that PSSNs tend to strengthen one’s emotional satisfaction with the current sanitation practice. Unlike material satisfaction, emotional satisfaction is independent of attained sanitation safety and likely to impair upward shifts in the sanitation ladder.

We believe that the distinction between material and emotional satisfaction and the outlined mechanism implying a potentially consequential role of the latter have a broader relevance for sanitation and other interventions utilizing the persuasive forces of social norms, often together with the social construction of risks. In addition to PSSNs, also the perception of health related risks and benefits were found to enhance emotional satisfaction with current sanitation practice. In the context of sanitation, these variables are to a large extent subject to the process of social construction. By contrast, the perception of non-health risks and benefits (including risks and benefits related to privacy, smell, cleanliness, comfort, and convenience), which is comparatively more determined by respondents’ own experience, and also the educational variables of factual sanitation and hygiene knowledge were unrelated to emotional satisfaction. As such, the later factors may be seen as less prone to the potential adverse influences on sanitation outcomes described above.

In regards to sanitation, the mechanism described above is particularly relevant for contexts similar to that of this study characterized by widespread but generally poor-quality and non-durable latrines induced through CLTSH and through subsequent formal and semi-formal sanctions used to reinforce the recently introduced practice of fixed-point defecation. Similar sanitation patterns were reported for different parts of Ethiopia [29,41,42,51] and elsewhere [27,31,32]. Socio-environmental vulnerability and infrastructural constraints interwoven with little effort to address both the supply and demand for upgrading existing sanitation facilities were other features symptomatic for the context of our study. All of these observations imply uncertain long term impacts of normative CLTS interventions if used largely as a stand-alone approach to sanitation safety change. Although this is arguably known to many practitioners from their field experience [52,53], the link between PSSNs and emotional satisfaction proposed and documented in this study provides a possible conceptual explanation.

As already mentioned, notable differences were identified between the roles of perceived health-related and non-health risks and benefits. The latter measure was a considerably stronger predictor of sanitation safety, while perceived health-related risks and benefits revealed a considerably stronger positive relationship with emotional satisfaction. These results are consistent with earlier evidence that motivators other than those that are health-related typically drive improvements to sanitation safety in households [5,9,15,16]. Here, we attempted to shed some new light on these differential effects. Inspired by the social amplification/attenuation of risk framework, we proposed that PSSNs can work as a social filter that can shape impacts of other independent variables of interest on the examined dependent variables. More specifically, PSSNs were shown to attenuate the effect of perceived health-related risks and benefits on sanitation safety and amplify its effect on emotional satisfaction. By contrast, the effect of perceived non-health risks and benefits on emotional satisfaction was significant only for observations with low PSSNs.

Sanitation behavior interventions make use of various inputs such as those targeting expectations about the opinions of others, information on the behavior of others, emotions and emotional experiences, perception of various risks and benefits, or knowledge about preventive or facilitating measures among others. Their results depend on whether and how effectively the right combinations of inputs are targeted. Our findings described above imply that attempts to construct the perception of health risks and benefits through sanitation interventions utilizing mechanisms of social influence would only have a limited impact on longer term improvements of sanitation safety. An emphasis on non-health risks and benefits is likely to generate better outcomes of normative/persuasive sanitation interventions.

The relationship between knowledge of sanitation and hygiene messages and the level of sanitation safety vanished when the PSSNs measure was included in the regression model. At the same time, PSSNs demonstrated a strong effect on sanitation safety and also a significant association with the knowledge of sanitation and hygiene messages. These results indicate that PSSNs play a mediating role through which some of the impacts of factual sanitation and hygiene knowledge on sanitation safety can be transmitted. Importantly, it means that sanitation outcomes can be seen as dependent upon the capacity of social influences (conformity with a norm, social networks and interactions etc.) to shape the hygiene and sanitation related awareness in desirable ways. These results underline the importance of educational and communicative components conditional to their integration into normative/persuasive sanitation strategies.

In general, the findings described above suggest that the mechanisms through which PSSNs influence sanitation outcomes depend on whether and how sanitation norms are internalized. The adverse effects are more likely to occur when compliance with a norm is externally determined through enforcement, negative sanctions but also when it is based on socially constructed symbolic risks rather than when it is driven by internal motivations (e.g., actually recognizable risks and benefits). The internalization of (sanitation) norms thus represents a key precondition for the long term sustainability of persuasive/normative interventions. In the context of the present article, sanitation norms were widespread but it was less clear to what extent they were internalized by the people.

Some limitations of this study have to be acknowledged. It is based on a mild sample size and cross-sectional data. As a result, statistical associations were established rather than cause-and-effect relationships in a strict sense. Further panel data research is also warranted to confirm the presumably negative link between emotional satisfaction with current sanitation practices and future upward shifts in the sanitation ladder. Although the link is intuitively reasonable, it has not been tested empirically in this paper which used cross-sectional data. Another important challenge to consider is the deployment of a more sophisticated measurement of sanitation safety that would account for the links between the parameters of sanitation safety examined in this study, the principal transmission pathways of fecal exposure, and the prevalence of infectious and parasitic diseases.

## 5. Conclusions

Advances in sanitation interventions have largely been driven by field-level experimental practices rather than theoretically [54,55]. Also, recent influential randomized counterfactual evaluations of sanitation interventions [27,28,56] have concentrated on the empirical examination of the outcomes of particular sanitation interventions rather than on testing their underlying factors and theories. On the other hand, the present study has attempted to add to the theoretical understanding of mechanisms operating beyond these interventions, which has unfortunately received little attention, relatively speaking.

Widespread perception of unacceptability of OD associated with a shared belief that individuals who defecate in the open pose a risk of disease for the whole community is thought to trigger the community’s desire for the elimination of OD practice, encourage mutual support, and propel the community into action. Therefore, constructing new PSSNs around the unacceptability of OD has been considered as a key element in improving sanitation outcomes at community level. However, to our knowledge, no prior research has empirically examined how PSSNs instilled through persuasive/normative sanitation interventions such as CLTS influence sanitation safety. This paper thus provides a first exploratory study focused in this direction. We proposed and analyzed how PSSNs interplay with other sanitation determinants such as the perception of risks and benefits associated with OD and latrine ownership, respectively, and factual sanitation and hygiene awareness. Three more general observations achieved in this study can be emphasized as follows. First, we proposed a potentially adverse feedback of PSSNs on sanitation safety via the effect of social conformity on emotional satisfaction, with the latter being conceptualized as the satisfaction with current sanitation practice which is independent of the functionality and durability of sanitation facilities. Second, drawing on the social amplification/attenuation of risks framework, we argued that the perception of social norms (stemming from the effects of social conformity, social networks and interactions etc.) is not only influential per se but also because it works as a social filter that shapes effects of other sanitation determinants targeted in interventions. Third, our findings underscore a key importance of norms internalization for sustaining sanitation outcomes. This explorative study offers a new understanding of the role of social norms in sanitation and more options for researching and designing sanitation interventions.

## Figures and Tables

**Table 1:** Questions and statements used to construct the composite score of PSSNs.

Question or Statement	Most Frequent Response
Do other people outside of your household in your village mostly defecate in a latrine?	“All” = 69%
Do you think that other people in your village should defecate in a latrine?	“Definitely” = 92%
People in your village think your family use latrine regularly	“Strongly agree” = 76%
If my neighbors use latrine, our family should use it as well	“Strongly disagree” = 67%
People in this village think you should use a latrine for defecation	“Strongly agree” = 76%

**Table 2:** Main variables of interest and their basic descriptive statistics (*N* = 368).

Variable	Shortened	Min	Max	Mean	SD
Sanitation safety (composite index, standardized)	Sanitation safety	−2.53	1.78	0.00	1.00
Satisfaction with current sanitation situation of own household (binary)	Satisfaction	0.00	1.00	0.72	0.45
Perceived social sanitation norms (composite index, standardized)	PSSNs	−3.09	0.75	0.00	1.00
Perceived health-related disadvantages of OD and advantages of private latrine (standardized)	Health-related risks and benefits	−2.03	2.82	0.00	1.00
Perceived non-health disadvantages of OD and advantages of private latrine (standardized)	Non-health risks and benefits	−2.06	3.08	0.00	1.00
Awareness about ways how to prevent diarrhea (binary)	Diarrhea prevention awareness	0.00	1.00	0.72	0.45
Hygiene and sanitation messages remembered (standardized)	Sanitation and hygiene messages	−2.09	2.73	0.00	1.00

**Table 3:** Predictors of sanitation safety.

Variables	Main Effects Model with PSSNs: Beta Coefficients (Standard Errors)	Main Effects Model without PSSNs: Beta Coefficients (Standard Errors)	Moderation by PSSNs: Beta Coefficients of Interaction Terms (Standard Errors) *R*^2^ of Models with Interaction Terms	Mediation through PSSNs: Indirect Effect (Standard Error)
PSSNs	0.302 (0.062) **	Excluded	Not relevant	Not relevant
Health-related risks and benefits	0.049 (0.052)	0.087 (0.053)	−0.146 (0.056) ** 0.402	Prerequisites not met
Non-health risks and benefits	0.169 (0.058) **	0.113 (0.061) *	0.059 (0.060) 0.388	Prerequisites not met
Diarrhea prevention awareness	0.270 (0.160) *	0.319 (0.163) *	0.025 (0.114) 0.386	Prerequisites not met
Sanitation and hygiene messages	0.115 (0.066)	0.218 (0.064) **	−0.072 (0.048) 0.391	0.103 (0.030) **
*R*^2^	0.386	0.325	-	-

Notes: ** Significant at the 0.01 level; * 0.05 level; *N* = 354; Robust standard errors reported; Controlled for village-level fixed effects, sex of household head, education and age of respondent, household size, source of drinking water, type of house, household income.

**Table 4:** Predictors of satisfaction with current sanitation practice (logistic regressions).

Variables	Main Effects Model with PSSNs: Beta Coefficients (Standard Errors)	Main Effects Model without PSSNs:Beta Coefficients (Standard Errors)	Moderation by PSSNs:Coefficients of Interaction Terms (Standard Errors) Nagelkerke *R*^2^ of Models with Interaction Term
PSSNs	0.781 (0.211) **	Excluded	Not relevant
Health-related risks and benefits	0.941 (0.210) **	0.916 (0.200) **	0.682 (0.222) ** 0.626
Non-health risks and benefits	0.496 (0.228) *	0.237 (0.206)	−0.578 (0.232) * 0.609
Diarrhea prevention awareness	−0.168 (0.512)	0.014 (0.493)	0.328 (0.405) 0.605
Sanitation and hygiene messages	−0.043 (0.211)	0.123 (0.196)	−0.163 (0.207) 0.605
Sanitation safety	1.741 (0.270) **	1.823 (0.263) **	−0.260 (0.322) 0.605
Nagelkerke *R*^2^	0.603	0.569	−

Notes: ** Significant at the 0.01 level; * 0.05 level. *N* = 350; Controlled for village-level fixed effects, sex of household head, age of respondent, and livestock ownership measured in tropical livestock units. Mediation models were not found relevant for any of the independent variables of interest.

## Appendix A

**Table A1:** Selected descriptive characteristics of the sample (*N* = 368).

Variables	Statistics
Age of respondents (SD)	40 years (13)
Female respondents	64%
Female headed households	19%
Household size (SD)	5.99 (2.06)
Average number of children under 5 years (SD)	0.73 (0.75)
Number of elderly above 50 years (SD)	0.41 (0.69)
Families with disabled persons	2%
Illiterate respondents	58%
Illiterate household heads	46%
Protestant religion (Orthodox religion)	87% (12%)
Farming as primary source of livelihood	96%
Household monthly income (calculated based on both in cash and in kind income) in Ethiopian Birr (SD)	647 (506)
Household land ownership in hectares (SD)	0.81 (0.68)
Household livestock ownership—number of oxen, bulls, and cows (SD)	2.43 (1.80)
Livestock ownership in Tropical Livestock Units (large cattle 0.8; smaller cattle 0.6; sheep and goats 0.1; donkeys 0.4; hens and chickens 0.01)	3.01 (2.07)
Households living in traditional house	53%
Average time needed to collect drinking water (including waiting time)—avg. dry and rain period (SD)	76 min (73)
Households using unprotected drinking water in both dry and rain period	30%

**Table A2:** Predictors of sanitation safety (full-specification of main effect model; i.e., including regression estimates for control variables).

Variables		Beta Coefficients	Standard Errors
Focal variables of interest in this study	PSSNs	0.302	(0.062) **
	Health-related risks and benefits	0.049	(0.052)
	Non-health risks and benefits	0.169	(0.058) **
	Diarrhea prevention awareness	0.270	(0.160) *
	Sanitation and hygiene messages	0.115	(0.066)
Control variables in this study	Household size	0.103	(0.062)
	If female household head	−0.385	(0.141) **
	Age of respondent	0.059	(0.059)
	If respondent illiterate	−0.011	(0.092)
	Log of household income	0.067	(0.056)
	If traditional house	−0.159	(0.112)
	If unprotected source of drinking water	−0.171	(0.287)
Pseudo *R*^2^	*R*^2^	0.386	

Notes: ** Significant at the 0.01 level; * 0.05 level; *N* = 354; Robust standard errors reported; Controlled for village-level fixed effects; Continuous variables standardized by z-score.

**Table A3:** Predictors of satisfaction with current sanitation practice (full-specification of main effect model; i.e., including regression estimates for control variables), binary logistic regression.

Variables		Beta Coefficients	Standard Errors
Focal variables of interest in this study	PSSNs	0.781	(0.211) **
	Health-related risks and benefits	0.941	(0.210) **
	Non-health risks and benefits	0.496	(0.228) *
	Diarrhoea prevention awareness	−0.168	(0.512)
	Sanitation and hygiene messages	−0.043	(0.211)
Control variables in this study	Sanitation safety	1.741	(0.270) **
	If female household head	−0.547	(0.458)
	Age of respondent	0.137	(0.211)
	Livestock ownership in Tropical Livestock Units	0.381	(0.204)
Pseudo *R*^2^	Nagelkerke	0.603	

Notes: ** Significant at the 0.01 level; * 0.05 level. *N* = 350; Controlled for village-level fixed effects.
